# Macrophages in the Aging Liver and Age-Related Liver Disease

**DOI:** 10.3389/fimmu.2018.02795

**Published:** 2018-11-30

**Authors:** Elizabeth C. Stahl, Martin J. Haschak, Branimir Popovic, Bryan N. Brown

**Affiliations:** Department of Bioengineering, Pittsburgh Liver Research Center, McGowan Institute for Regenerative Medicine, University of Pittsburgh, Pittsburgh, PA, United States

**Keywords:** Kupffer cell, monocyte-derived macrophage, chronic liver disease, inflammation, fibrosis, senescence, mitochondria

## Abstract

The number of individuals aged 65 or older is projected to increase globally from 524 million in 2010 to nearly 1. 5 billion in 2050. Aged individuals are particularly at risk for developing chronic illness, while being less able to regenerate healthy tissue and tolerate whole organ transplantation procedures. In the liver, these age-related diseases include non-alcoholic fatty liver disease, alcoholic liver disease, hepatitis, fibrosis, and cirrhosis. Hepatic macrophages, a population comprised of both Kupffer cells and infiltrating monocyte derived macrophages, are implicated in several chronic liver diseases and also play important roles in the homeostatic functions of the liver. The effects of aging on hepatic macrophage population dynamics, polarization, and function are not well understood. Studies performed on macrophages derived from other aged sources, such as the bone marrow, peritoneal cavity, lungs, and brain, demonstrate general reductions in autophagy and phagocytosis, dysfunction in cytokine signaling, and altered morphology and distribution, likely mediated by epigenetic changes and mitochondrial defects, that may be applicable to hepatic macrophages. This review highlights recent findings in macrophage developmental biology and function, particularly in the liver, and discusses the role of macrophages in various age-related liver diseases. A better understanding of the biology of aging that influences hepatic macrophages and thus the progression of chronic liver disease will be crucial in order to develop new interventions and treatments for liver disease in aging populations.

## Introduction to macrophages in the liver

The liver is an important immunological organ, serving as a surveillance system for gut-derived pathogens and producing several key immune components—complement, acute phase, and coagulation proteins (1, 2). While hepatocytes and certain non-parenchymal cells possess some inherent immunological properties [see reviews 3 and 4)], multiple populations of CD45^+^ immune cells are transiently or permanently located in the liver.

The healthy liver is home to several populations of lymphocytes, including natural killer (NK) cells, NK T-cells, B-cells, mucosal associated T-cells and γδ-T-cells (5–8). In humans, 40% of the resident lymphocyte population are composed of NK cells, while in mice 40% of the resident lymphocytes are NK T-cells.

Innate immune cells also have a large presence in the liver. The majority of immune cells in the liver are myeloid derived cells (9). Dendritic cell (DC) populations, including both myeloid and plasmacytoid DCs, are present in the liver and can activate T-cells under appropriate conditions (10). Myeloid-derived suppressor cells (MDSC) are also present and suppress T-cell activation (11). Macrophages derived from circulating monocytes have been identified as a motile population of myeloid cells infiltrating the liver during inflammation and at varying levels during homeostasis (12–14). The resident macrophage, known as the Kupffer cell (KC), is the most highly represented immune cell, comprising nearly one-third of non-parenchymal cells in the liver (15).

Macrophages play a key role in the homeostatic functions of the liver as well as in disease states and are among the most widely studied immune cells in the liver (15). The prevalence and severity of many chronic liver diseases increases with age, including alcoholic hepatitis, non-alcoholic steatohepatitis, cirrhosis, and hepatocellular carcinoma (16). However, mechanisms underlying changes in the liver structure and cellular function, including hepatocytes and resident immune cell populations, are not well understood. This review will cover the developmental origin and function of macrophages in the liver, as well as their implications in several age-related liver diseases, in order to better understand and expose gaps in the knowledge of the biology of liver aging and disease. As most of the experimental data has been collected from murine animal models, this review focuses largely on preclinical studies in mice and extends these findings to chronic liver disease in humans.

### Development and polarization of macrophages

Macrophage development begins in the extraembryonic yolk sac from erythro-myeloid progenitors, a process known as primitive hematopoiesis, prior to the appearance of hematopoietic stem cells or monocytic precursors (17, 18). After this transient wave of primitive hematopoiesis, hematopoietic stem cells appear in the aorto-gonado-mesonephric region and migrate to the fetal liver, marking the start of definitive hematopoiesis and development of naïve macrophages through monocytic precursors (17). Definitive hematopoiesis remains in the fetal liver until approximately E18 in mice or 12 weeks post-conception in humans, after which hematopoietic stem cells migrate from the fetal liver to the bone marrow niche, where they will remain throughout adulthood (19).

Macrophages can be subdivided into tissue resident or monocyte derived populations. The number of resident macrophages varies considerably based on the tissue, and approximately 80% of total tissue resident macrophages are located in the liver (20). Resident macrophages can be colonized from a single developmental source, such as microglia and Kupffer cells, which are derived from yolk sac primitive macrophages. Alternatively, resident macrophages may be repopulated several times during development, such as Langerhans cells in the skin, which are originally derived from yolk-sac macrophages and later replaced by fetal liver monocytes (13, 21). Macrophage populations in the gut, spleen, and lungs are continuously replenished by monocyte input throughout adulthood (12, 13).

During inflammation, both resident and monocyte-derived macrophages participate in migration, expansion, and signaling depending on the tissue type and stimulus. Resident tissue macrophages, including Kupffer cells, peritoneal macrophages, and pleural macrophages, have been shown to undergo rapid proliferation in response to Th2 cytokines, such as interleukin-4 (22). Infiltrating macrophages can be derived from either classical (CD14^+^CD16^−^ human, Ly6C^hi^ mouse) or non-classical (CD14^+^CD16^+^ human, Ly6C^lo^ mouse) monocytes, which extravasate into tissue sites under chemokine concentration gradient guidance (17, 23). Following extravasation, monocytes differentiate into macrophages whose functionality are largely based on the integration of the various signaling molecules present in the local microenvironment (24, 25).

**Figure 1:**
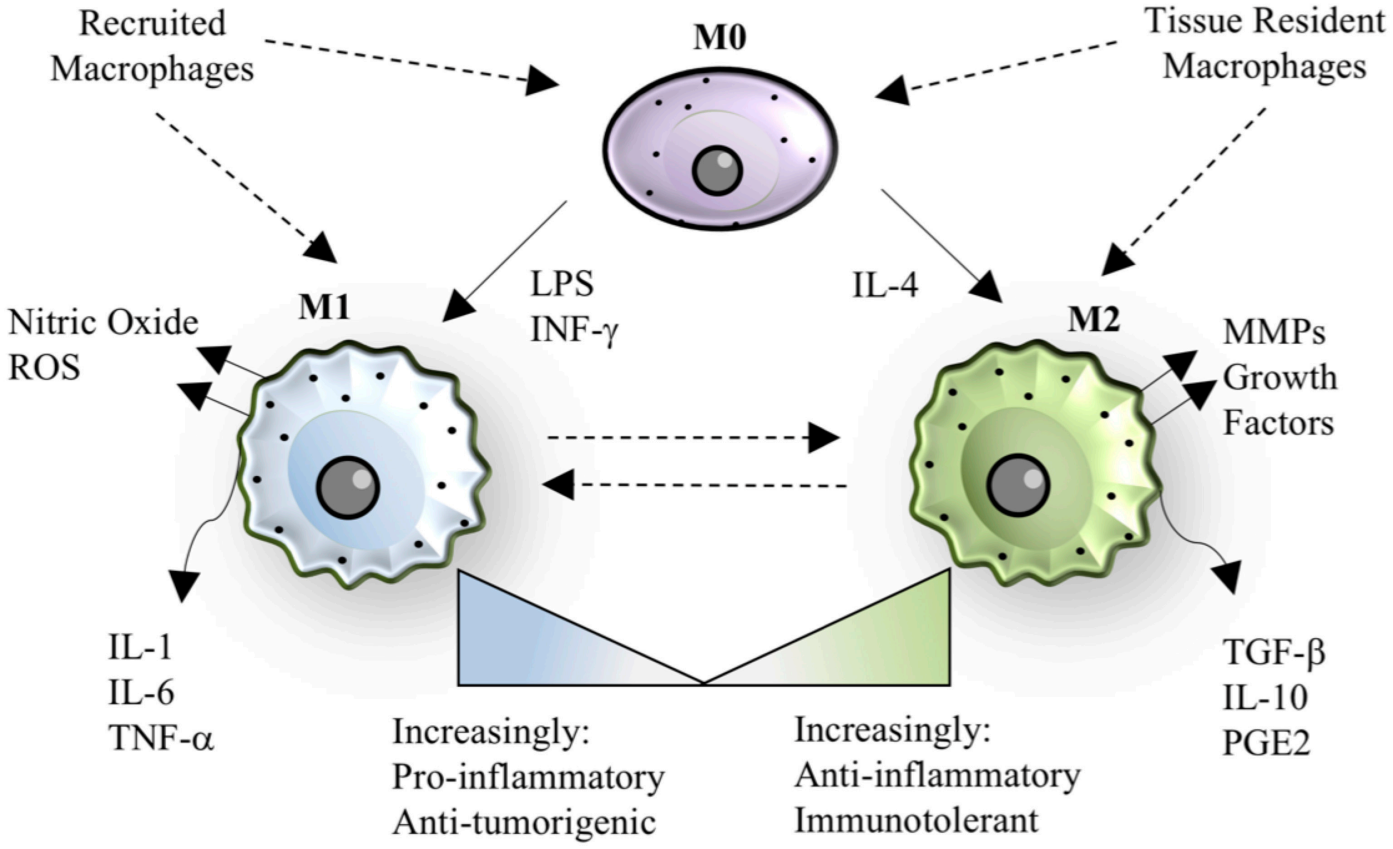
Macrophage polarization. The paradigm of macrophage polarization begins with a quiescent or patrolling M0 cell, that may be derived from either circulating monocytes or tissue-resident populations. The transition from M0 to M1 can be stimulated by lipopolysaccharide (LPS) or interferon-gamma, resulting in a cell that has pro-inflammatory and anti-tumorigenic properties. M1 macrophages also produce nitric oxide and other reactive oxygen species (ROS). The transition from M0 to M2 can be stimulated by interleukin-4, resulting in a cell that has anti-inflammatory or immunotolerant properties. M2 macrophages also produce matrix metalloproteinases (MMPs) and growth factors that aid in tissue regeneration. M1 and M2 phenotypes are dynamic and exist on a spectrum, where recruited macrophages tend to be more “M1-like” in the case of bacterial infection, and tissue-resident macrophages tend to be more “M2-like” during homeostasis. In specific instances, such as helminth infection, recruited macrophages take on an M2 phenotype, demonstrating the versatility and adaptability of the macrophage polarization spectrum in response to the signals in a given context.

In addition to developmental heterogeneity, macrophages are extraordinarily dynamic cells that exhibit various phenotypes ranging from a pro-inflammatory, classically activated “M1” polarization state to an anti-inflammatory, alternatively activated “M2” polarization state, as shown in Figure 1 (26, 27). M1/M2 polarization refers to extreme phenotypes that can be simulated *in vitro* with the addition of various stimuli (lipopolysaccharide and/or interferon-gamma for M1 activation; interleukin-4 for M2 activation), but the scenario *in vivo* is often more complex and thus polarization is a spectrum of phenotypes, including but not limited to M2a, M2b, and M2c sub-groups (27, 28).

In general, M1 polarization leads to production of canonical pro-inflammatory cytokines (IL-1, IL-6, IL-12, IL-18, TNF, IL-23, IL-27), reactive oxygen species such as nitric oxide, and chemokines (CCL2, CCL5, CXCL9, CXCL10, CXCL11, IL-8) (27). Macrophage pro-inflammatory cytokine maturation can occur through inflammasome-independent or inflammasome-dependent mechanisms (29, 30). Inflammasome formation is induced by damage-associated molecular patterns (DAMPs) and pathogen-associated molecular patterns (PAMPs) binding to nod-like receptors, including NLRP3 and NLRP1, or to Pyrin receptor family members such as Pyrin and AIM2 on the surface of macrophages (31). Docking proteins, including apoptosis-associated speck-like protein, assemble and recruit pro-caspase 1, where proximity-based autoproteolytic cleavage catalyzes the activation of caspase-1 (29, 32) Caspase-1 activation subsequently catalyzes the cleavage of the pro-forms of IL-1β and IL-18 into activated forms. Caspase-1 may also induce pyroptosis, a programmed cell death pathway characterized by cellular lysis and endogenous DAMP release into the environment, to induce additional inflammatory cell recruitment (33).

M2-polarized macrophages typically appear following M1-polarized macrophages in an injury setting and serve as a counterbalance to resolve inflammation and promote tissue repair (34). The M2 polarization state leads to secretion of cytokines commonly associated with anti-inflammatory properties, such as IL-10, as well as various matrix-modulatory factors including transforming growth factor-β (TGF-β) and matrix metalloproteases (MMPs) (35). The overproduction of remodeling growth factors can lead to excessive deposition of matrix proteins (i.e., fibrosis), or excessive angiogenesis and immunosuppression in the case of tumor-associated macrophages (TAMs) (36). Therefore, the temporal regulation of M1 and M2 macrophage responses are extremely important for appropriate outcomes following injury and infection and are often dysregulated in chronic disease.

### The origin and function of the kupffer cell

The Kupffer cell is a primitive cell, appearing early during embryogenesis (E9.5-E12.5 in mice), derived primarily from the yolk sac (14, 37, 38). Importantly, the KC population is maintained through self-proliferation, with minimal input from circulating monocytes during homeostasis (12–14, 22).

The tissue-resident Kupffer cell is the key detector of commensal or pathogenic microbial signals, danger signals, and tumor cells moving through the hepatic circulation (15, 39, 40). KCs are located along the hepatic sinusoids allowing for the low-pressure blood supply come into contact with both KCs and hepatocytes through the fenestrated endothelium (2). KCs express the complement receptor CR1g, which binds complement fragments C3b and iC3b, allowing phagocytosis of complement C3-opsonized particles even under low-pressure blood flow (41, 42). Bacterial clearance by KCs is crucial for host defense as 80% of blood-borne bacteria accumulate in the liver and are destroyed there (15, 39, 40).

PAMPs and DAMPs are present in relatively high concentrations in blood entering the liver from the gut, via the portal vein, and engage with pattern recognition receptors (PRRs), including Toll-like receptors, on the surface of macrophages and hepatocytes (43–45). With low levels of bacterial endotoxins, KCs promote immune tolerance by secreting anti-inflammatory factors including IL-10, TGF-β, and prostaglandin-E2 (PGE2), thereby inducing regulatory T-cells (46, 47). In the presence of higher concentrations of damage or pathogen-associated signaling molecules, KCs can become polarized toward an M1 phenotype and produce a variety of inflammatory cytokines including IL-1, IL-6, IL-12, TNF-α (15, 39, 40). Several liver diseases are influenced by KC activation and expansion, but their individual role has been difficult to dissect from more recently identified macrophage populations in the liver.

### The origin and function of other hepatic macrophage populations

Within the past decade, the heterogeneity of hepatic macrophages, i.e., Kupffer cells and monocyte-derived macrophages (mMØs), has become an emerging topic in hepatology. Fate tracing experiments in the brain first determined that resident microglia are established prenatally and maintained independently from monocyte input, which was later translated to the liver (48). Holt et al. were among the first to identify two populations of macrophages in the liver (49). Through bone marrow chimera experiments, cells expressing F4/80^hi^CD11b^lo^ were identified as KCs, while cells expressing F4/80^lo^CD11b^hi^ were found to be derived from circulating monocytic progenitors (49). Shortly after, Klein et al. classified two macrophage populations in the liver: KCs as immobile, “sessile” macrophages and mMØs as motile cells (50).

In most cases, murine mMØs in the liver are derived from an influx of bone marrow derived, Ly6C^hi^ monocytes, primarily driven by monocyte chemoattractant protein, which can be produced by KCs, stellate cells, or hepatocytes (also known as CCR2-CCL2 interactions) (51, 52). Secondary pathways of monocyte recruitment to the liver are CXCR3-CXCL10, CCR1-CCL5, and CCR8-CCL1 dependent (53–55). Murine mMØs may also be derived from Ly6C^lo^ monocytes trafficking from the spleen, which express CD11b, but are thought to take on a more patrolling or regulatory macrophage phenotype (56). Phagocytes from the peritoneal cavity, which express F4/80, CD11b, and GATA6, have been shown to cross into the liver after subcapsular liver lesion (57), but it remains unclear if this infiltration occurs in other liver injury settings.

Kinoshita et al. determined that murine mMØs and KCs could be distinguished by CD11b and CD68 expression, respectively (40). The CD11b^hi^ mMØs were radiosensitive and particularly efficient at producing IL-12, protecting the host against tumor xenografts, while the CD68^hi^ KCs were radioresistant and highly efficient at phagocytosis, protecting the host against bacterial challenge (40). The use of these differentiating markers has been expanded to several disease models, identifying a damaging role of TNF/FasL production by CD11b^+^ mMØs in carbon-tetrachloride acute liver injury and hepatitis in hypercholesterolemic mice (58, 59). The CD11b^+^ mMØs were also found to accumulate in the liver following repeated lipopolysaccharide injections, but suppressed TNF efflux into the systemic circulation, thereby reducing lethal septicemia (60). In addition, the CD11b^+^ mMØs were recruited during diet-induced steatohepatitis in FGF5 deficient mice and following partial hepatectomy, where they were crucial for liver regeneration (61, 62). Others have observed that monocyte derived cells protect the liver from iron toxicity in hemolytic anemia by ingesting senescent and dying erythrocytes (63).

More recently, the C-type lectin, Clec4f, has been identified as a selective murine KC marker (64). Interesting, ablating KCs via Clec4f-driven diphtheria toxin causes an influx of mMØs, which can differentiate into nearly identical KCs when this “niche” is made available. Only 12 genes remained differentially expressed between monocyte derived KCs and embryonically derived KCs, including *CD209f, CD163, C2, CCR3, Timd4*, and *Snrpn* at the time points examined (64).

Taken together, KCs and mMØs play heterogenous roles in various liver disease states and cannot be considered wholly harmful or beneficial without deciphering the given context. Importantly, infiltrating monocytes may also differentiate into dendritic cells, which play distinct immunostimulatory and antigen presentation roles in the liver (65). The heterogeneity of hepatic macrophages is less well defined in humans as compared to murine animal models. The role of these diverse hepatic macrophage populations in a given disease context is just beginning to be understood and has not yet been examined in the natural aging process, a major risk factor for several chronic liver diseases.

## Cellular manifestations of aging on hepatocytes and macrophages

The process of aging is closely associated with a number of degenerative modifications in the liver, where hepatic structure and cell function are observed to decline. Both hepatocytes and macrophages exhibit deficits in mitochondrial function, linked to a decline in autophagy and production of pro-inflammatory molecules (66, 67). While the effects of aging on hepatic macrophages have not been well characterized on a cellular and molecular level, several studies have examined macrophages from alternative tissue sources and may offer some insight about hepatic macrophages.

### Changes in hepatic structure and hepatocyte function

Several studies have shown that the volume of blood in the liver decreases in elderly individuals, leading to a total volume loss of 20–40% (68). In addition, the thickness of liver sinusoidal endothelial cells fenestrations increases, limiting the exchange of molecules to and from the liver (69). At the serum level, aging is associated with reductions in albumin and bilirubin, increases in alkaline phosphatase, and minimal changes in aminotransferase levels (70). The metabolism of cholesterol in the liver also decreases, leading to increased blood cholesterol and neutral fat levels over time (70). However, recent investigations suggest the most essential change in liver aging is loss of the functional liver cell mass (71).

Changes in the morphology of hepatocytes may be related to increased polyploidy, accumulation of lipofuscin in the cytoplasm, and declining surface area of endoplasmic reticulum and number of mitochondria, ultimately negatively affecting the function of hepatocytes (66, 72, 73). The decline in hepatocyte mitochondrial function has been suggested to enhance the vulnerability of aged livers to acute injury and to cause delays in liver regeneration (74). The oxidative capacity of the liver also declines with aging, and therefore medications that require oxidation, such as benzodiazepines, may accumulate in toxic levels and are not prescribed to elderly individuals (75). In addition, aging livers are known to accumulate a multiprotein C/EBPalpha-Brm-HDAC1 complex that silences elongation factor 2 (E2F)-dependent promoters, thereby reducing proliferation and regenerative capacity of hepatocytes (76).

Senescent cells accumulate in the liver during aging and in chronic liver disease; and may include hepatocytes, cholangiocytes, stellate cells, and immune cells [reviewed here: (77)]. Cells become senescent as a result of replicative exhaustion (telomere shortening), DNA damage, or oxidative stress, among other mechanisms (78). Senescent cells are characterized by the expression of cell cycle inhibitors p21, p16, and p53, which prevent replication and apoptosis, as well as secretion of pro-inflammatory cytokines that signal for their removal but can lead to tissue damage when chronically expressed (79, 80). Senescent hepatocytes accumulate with age and appear as an almost “universal phenomenon” in chronic liver diseases including hepatitis B and C infection, alcoholic and non-alcoholic liver disease, and genetic haemochromatosis (77). Senescent hepatocytes undergo metabolic changes, such as increased transport of conjugated bilirubin into the hepatic sinusoids and insulin resistance through dysregulated glucose transporter expression and Akt signaling among others, which dysregulates normal function (81). Interestingly, the length of telomeres was found to be conserved in aged hepatocytes and bile duct cholangiocytes but decreased in aged Kupffer cells and stellate cells, suggesting cell-specific mechanisms of senescent phenotype acquisition in the liver (82).

Overall, the effects of aging on non-parenchymal cell subsets, particularly hepatic macrophages, is an understudied area. Hilmer et al. determined that the number and basal activity of Kupffer cells was increased with old age in a rat model (83), however this work was performed before the heterogeneity of liver macrophages was fully contemplated. Similarly, Singh et al. identified a change in liver macrophage distribution with aging, where old mice had more F4/80^+^ macrophages located in large lymphoid structures (also containing T-cells and B-cells), and a reduction in the number of spindle shaped macrophages throughout the parenchyma (84).

Recent work by Maeso-Diaz et al. characterized molecular changes in LSECs, stellate cells, and hepatic macrophages in the livers of aged rats (85). The authors found a significant deregulation of LSEC phenotype with aging as demonstrated by downregulation of vasodilatory pathways (nitric oxide, heme oxygenase), increase in oxidative stress, and decrease in angiocrine markers (stabilin-2, CD32b, and VEGFR2), in addition to increased portal pressure and vascular resistance (85). Aged stellate cells had increased markers of activation (alpha smooth muscle actin and collagen I), increased intracellular lipid stores, and alterations in retinoid metabolism (85). Finally, the authors noted an increase in recruitment of proinflammatory cells in aged livers, including increased IL-6 expression in isolated hepatic macrophages, but no change in the traditional macrophage polarization markers (85). Human livers showed similar trends in downregulation of endothelial markers, but substantial work remains to extend and decode these findings in both aged murine and human systems.

### Declines in cell intrinsic macrophage function with aging

**Figure 2:**
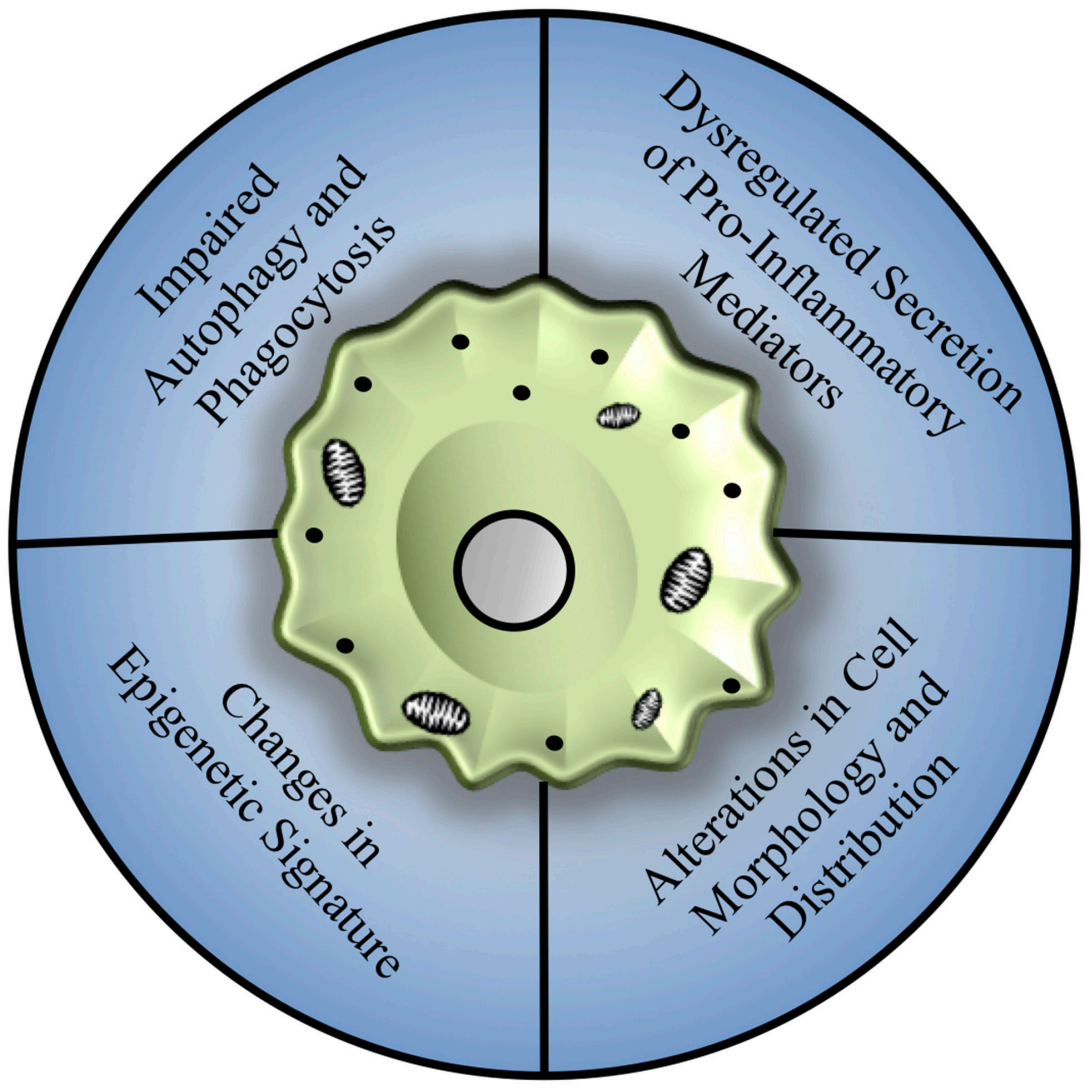
Mechanisms of age-associated decline in macrophage function. Macrophages from various tissue sources, including the bone marrow, peritoneal cavity, lungs, and brain, show an array of dysfunction including a general reduction in autophagy and phagocytosis, dysregulation of pro-inflammatory cytokine production associated with changes in toll-like receptor expression, and alterations in cell morphology and distribution that may be related to repopulation of tissues by infiltrating monocyte populations, largely mediated by changes in epigenetic signature and mitochondrial dysfunction.

The process of aging has been shown to affect macrophage polarization and function in multiple tissues and disease models, with compelling underlying mechanisms that may be broadly applicable to hepatic macrophages, as summarized in Figure 2.

Autophagy, or the intracellular degradation of damaged proteins and organelles in autophagosomes, is one such process which has been shown to become dysregulated with increasing age in macrophage populations (86). Deletion of autophagy genes *Atg5* and *Atg7* in bone marrow-derived macrophages results in decreased antigen presentation capacity, impaired maturation, altered mitochondrial metabolism, downregulation of surface receptors such as toll-like receptor 4 (TLR4), and increased secretion of pro-inflammatory cytokines (86, 87). The phenotype of autophagy deficient bone marrow macrophages closely mimics the phenotype of macrophages isolated from aged individuals (86), which may be driven by the hypermethylation of autophagy associated genes (88). It remains to be seen if restoring autophagic flux to aged macrophages can improve their function and polarization status, however caloric restriction has been shown to improve both longevity and autophagic capacity in animal models (89).

Phagocytic clearance of extracellular pathogens and antigen presentation tends to be attenuated in aged macrophage populations as well (90). While no difference in phagocytic capacity was found between young and aged bone marrow derived macrophages, peritoneal macrophages exhibit an age-associated decline in phagocytic capacity and antigen presentation (90). This reduction in phagocytic ability was driven by age-related alterations in the local microenvironment, as young peritoneal macrophages transplanted into aged peritoneal space exhibited reduced phagocytic and antigen presentation capabilities (90). Alveolar macrophages isolated from aged individuals have been shown to exhibit reduced phagocytic capacity as well as attenuated expression of genes associated with macrophage proliferation (91). This reduction both in cell number and phagocytic ability contributes to the age-related mortality risk following pathogen infection, such as in the case of an influenza lung infection model (91). In the liver, there have been mixed reports on the maintenance of Kupffer cell phagocytosis, where one study reported a deficit (92) and another reported an increase in phagocytosis with aging (83).

Furthermore, the reduction in expression levels of autophagosome components prevents the endocytosis of both inflammasome components and damaged mitochondria and can thereby promote chronic activation of pro-inflammatory signals in aged cells (87). This phenomenon has been termed “inflamm-aging,” one of the hallmarks of aging, and has been strongly correlated to morbidity (93). In addition to dysregulated cytokine production, aging can promote alterations in the secretion of oxidative species (94–96). Bone marrow-derived macrophages isolated from aged murine and human donors have been shown to produce greater concentrations of nitric oxide and reactive oxidative species than macrophages isolated from young donors (96). However, this upregulation in the secretion of reactive oxidative species was not observed following pro-inflammatory stimulation of peritoneal macrophages (94, 95). Further studies will be necessary to fully understand tissue-specific changes in respiratory burst characteristics.

The morphology of macrophages can also be influenced by the aging process. Microglia have been shown to increase cell soma volume while reducing the length of cellular processes in aged brains (97). These morphological changes reduce the capability of the microglia to interact with neural cells and perform routine surveillance of the local microenvironment (97). While microglial population sizes increase to account for this reduction in cell process size (17), the population expansion tends to occur in a non-homogenous manner, disrupting the uniform microglial distributions commonly observed in young animals (97). In addition, aged microglia exhibit enhanced secretion of reactive oxidative species following central nervous system injury through upregulation of NADPH oxidase 2 (NOX2) (98), and secrete elevated concentrations of IL-1β mediated by hypomethylation of CpG sires in the IL-1β proximal promoter (99).

Infiltration of monocyte-derived macrophages to multiple tissue sites has been observed in age-related diseases, including Alzheimer's disease (100), which leads to increased inflammation and phagocytosis. In the cardiovascular system, the tissue resident macrophage population (yolk-sac derived, M2-like) is replaced by bone marrow-derived macrophages over time (101, 102). This shift in macrophage populations contributes to deleterious outcomes following cardiac injury, such as chronic inflammation, fibrotic scar deposition, and reduced re-vascularization of ischemic tissue following injury (101, 103). It remains unclear whether age-related changes in macrophage responses are a primary result of shifting cell origins and polarization states driven by the microenvironment or by cell intrinsic functions, although both are likely in play.

### The role of the aged microenvironment on macrophage function

While cell intrinsic properties, such as mitochondrial capacity and autophagy, may drive changes in age-associated macrophage polarization, this only represents a partial view of the complex innate and adaptive immune dysfunction which occurs with age. For example, age-related alterations in the T cell compartment, which are numerous [reviewed here (104, 105)], can shift the relative Th1 and Th2 cytokine concentrations and directly impact macrophage polarization (106, 107).

Non-immune cell types can also contribute to the age-associated immune dysfunction. With age, increasingly large numbers of pre-adipocytes differentiate into mature adipocytes, with enhanced secretion of leptin, TNF-α, and IL-6, and reduced secretion of adiponectin—a factor commonly associated with M2 macrophage polarization, thereby promoting M1 phenotypes (108–110). Numerous DAMPs, which have been shown to promote inflammasome formation and activation, also have age-related increases in concentration, including cholesterol (111), amyloid-β (112, 113), hydroxyapatite crystals (114), purine catabolic end products such as uric acid (115), and ATP (116). Radical oxidative species, which tend to exhibit an increase in production and secretion with increasing age, have also been shown to play an important role in inflammasome activation through oxidation of proteins, lipids, and DNA, leading to inflammatory cytokine secretion into the aged microenvironment (29, 117, 118). More recently, the effect of the aging gut microbiome on macrophage function has been considered (119). Raising mice under germ-free conditions preserves the bactericidal capacity of alveolar macrophages and reduces secretion of IL-6 in old age, mediated by reduced dysbiosis and improved gut permeability (119).

While this is only a brief consideration of additional factors that have been observed to influence macrophage function, it serves to highlight the complexity and tissue specificity that defines age-related immunomodulation. The mechanisms governing the acquisition of age-related dysfunction in infiltrating and tissue resident macrophage populations continues to be an active area of research, and it will be essential for investigators to utilize an array of cell markers and descriptive methods to appropriately place cells along the spectrum of polarization states.

## The role of macrophages in age-related liver disease

**Figure 3:**
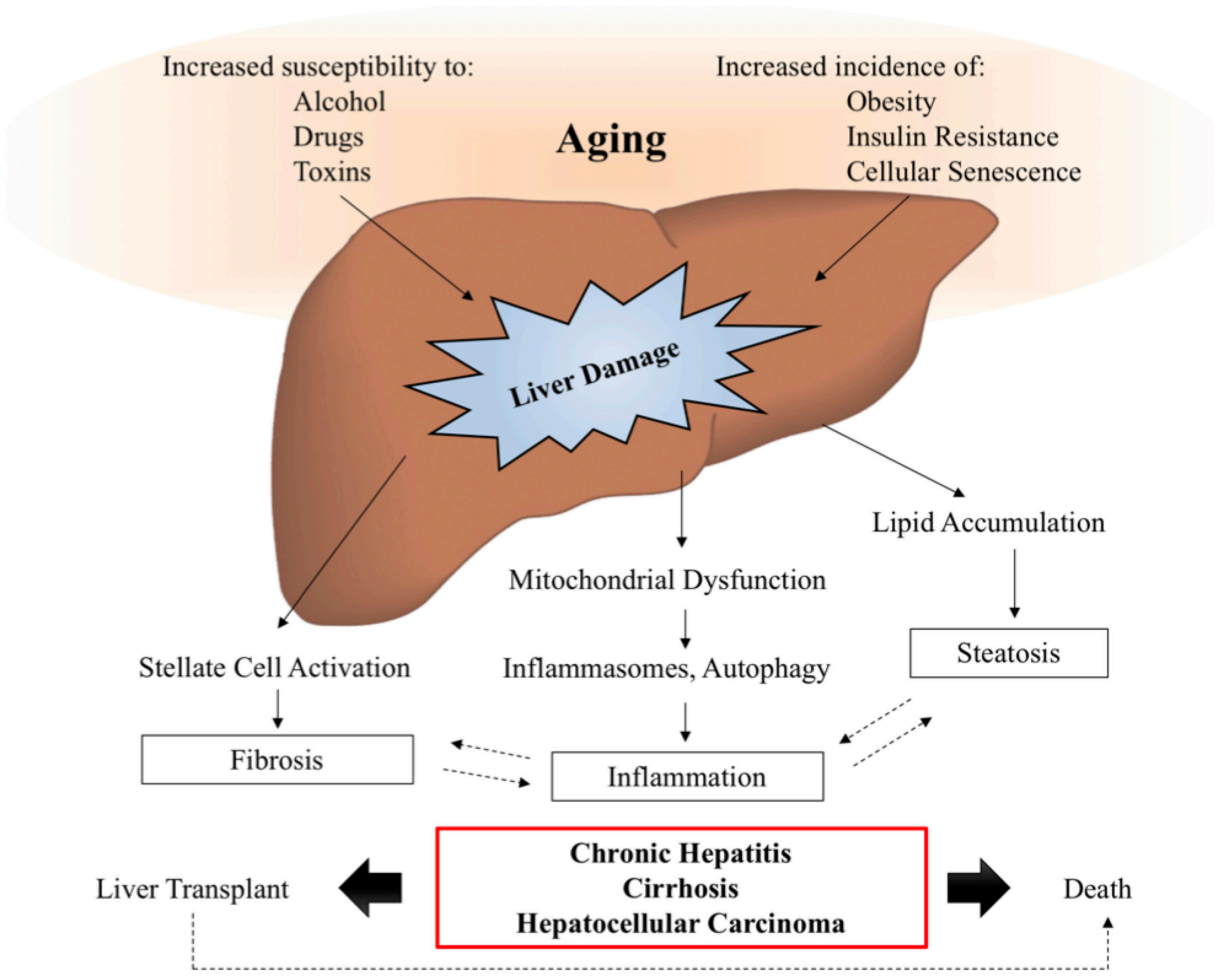
Aging and liver disease. With increasing age, the liver becomes more susceptible to damage from alcohol, drugs, and toxins, while the prevalence of metabolic disease, obesity, and cellular senescence are known to increase. These insults lead to the activation of signaling pathways driving liver pathology, such as inflammation, steatosis, and fibrosis, which are often involved in positive feedback loops, further exacerbating the symptoms of liver disease. In a subset of patients, liver disease will progress to chronic hepatitis, cirrhosis, and hepatocellular carcinoma, also known as end stage liver disease, for which transplant is the only viable therapy. Studies suggest that elderly patients have decreased survival post-transplantation, demonstrating the need for additional therapies to intervene and prevent the onset of age-related liver disease symptoms.

Aging is a major risk factor for the development and prognosis of several chronic liver diseases and conditions, including nonalcoholic fatty liver disease, alcoholic liver disease, hepatitis C, and an increased susceptibility to develop fibrosis and cirrhosis (16). The reduced capacity of aged livers to regenerate and to tolerate transplantation leads to increased risk of mortality from chronic liver diseases, as demonstrated in Figure 3.

Both Kupffer cells and infiltrating monocytes are implicated to various levels in the etiology of chronic liver disease, however the distinction between these two cell populations is less clear in human clinical liver samples compared to animal studies. Importantly, most animal models of chronic liver disease are induced in young, healthy animals through genetic manipulation or by administering specific diets or chemical toxins. Findings from these animal studies are important to identify novel mechanisms regulating liver disease but may not prove relevant or all-encompassing when translating the findings to older, human populations, as evidenced by the moderate success of several anti-fibrotic therapies in clinical trials (120). Distinguishing between the biology of aging and age-associated pathologies can be a difficult task but is an important effort in the field of hepatology. Additional studies are needed to assess the effect of aging on the liver prior to the onset of age-associated pathologies in order to dissect mechanisms of aging from the manifestation of the pathology itself.

### Liver fibrosis

Aging is a major risk factor for the progression of liver fibrosis, particularly in hepatitis, which can advance to portal hypertension and cirrhosis (121). Fibrosis occurs when excessive connective tissue is deposited following acute or chronic liver damage, and is often the starting point of architectural distortion and dysfunction that prevents normal functioning of the liver (122). Age-related dysfunction in hepatic macrophages and stellate cells are heavily implicated in development of fibrosis and may be related to oxidative stress and macrophage polarization, but these mechanisms are not well understood (123).

In general, an increase in M1-polarized macrophages appears at the early stages of liver disease and promotes or exacerbates fibrosis, cirrhosis, and eventual liver failure. In clinical studies, the progression from hepatitis to fibrosis and eventually cirrhosis was associated with enrichment of CD14^+^CD16^+^ non-classical monocytes in the liver, which have been shown to activate stellate cells *in vitro* based on TGF-beta release (124). These cells may be derived from classical CD14^+^CD16^−^ monocytes or infiltrate directly (125). Activation of the CCL2-CCR2 axis is associated with monocyte infiltration in both rodent models and patients with fibrosis and liver disease (126). A recent study found that the acquisition of M1 phenotypes in liver fibrosis may be regulated by interferon regulatory factor 5 (IRF5), which is significantly induced in liver macrophages in both mouse and human subjects developing fibrosis, and may represent a novel target for therapeutic intervention (127).

### Alcoholic liver disease

Elderly individuals are more likely to feel socially isolated and depressed, resulting in a rising alcohol consumption rate and prevalence of alcoholic liver disease in this population (128). Alcohol and its breakdown products are toxic to the liver, which can cause a disorder in lipid metabolism by increasing the synthesis of fatty acids and suppressing mitochondrial β-oxidation (129). The risk of alcohol toxicity is further increased in elderly populations due to alterations in metabolism or by the intake of certain medications (130).

Alcoholic liver disease can be classified into three stages: accumulation of extra fat in the liver (steatosis), alcoholic hepatitis, and cirrhosis. Macrophages were found to increase in both the early and late stages of alcoholic liver disease. Increased gut permeability and high levels of endotoxin in the portal blood results in activation of Kupffer cells (131). In addition, Ly6C^+^ macrophages were found to accumulate in murine models of alcoholic liver injury, regulated by CCL2 chemokine signaling (132). Clinical symptoms of alcoholic liver disease among the elderly are similar to younger counterparts, yet the prevalence of advancing to irreversible stages of the disease is greater in older individuals (133). Oxidative stress and production of TNF by hepatic macrophages, as a result of steatosis or leaky gut, causes progression to later stages (130). Furthermore, the expression of M1 pro-inflammatory genes was found to be higher in peritoneal macrophages from patients with alcohol-related cirrhosis and ascites compared to hepatitis-C related cirrhosis, suggesting systemic differences in macrophage polarization (134). Reducing leaky gut and inflammation have been identified as potential therapeutic targets for treating alcoholic liver disease (135).

### Non-alcoholic fatty liver disease

Non-alcoholic fatty liver disease (NAFLD) and non-alcoholic steatohepatitis (NASH) are leading causes of chronic liver disease globally and are projected to become the leading indication for liver transplantation in the United States (136–138). NAFLD often presents with metabolic syndrome, such as obesity and excessive visceral fat, hyperlipidemia, hypertension, insulin resistance and an increased secretion of pro-inflammatory cytokines (139). All of these components of metabolic syndrome are generally observed in elderly populations and many studies find increased rates of NAFLD among elderly people compared with their younger counterparts (140–142).

The mechanism for developing age-related steatosis is not fully understood but has been attributed to hepatocyte senescence driving a reduction in mitochondrial metabolism (143), decreased transport of insulin across the sinusoidal endothelium (144), reduction in autophagic flux (145), or chronic low-level inflammation (146, 147), leading to the build-up of toxic free fatty acids in the liver. Lifestyle choices, such as high fat diet (HFD), may also lead to the development of NAFLD in aged individuals. Work by Fontana et al. found that mice were equally susceptible to steatosis following HFD regardless of age. However, the older mice exhibited more severe hepatocellular injury and inflammation following administration of HFD that was attributed to increased M1 macrophage polarization in both the liver and white adipose tissues (148).

As in other chronic liver diseases, macrophages accumulate in the livers of NAFLD patients (149). Experimental mouse models have shown an accumulation of Ly6C^+^ monocytes is a critical step in the development of progressive fibrosis from steatohepatitis in a CCL2-dependent mechanism (131). In addition, lipids and free fatty acids contribute to DAMP-induced Kupffer cell activation (150). Patients with low-grade steatosis were found to have higher mRNA expression of M2 markers, CD206 and CD163, compared to patients with advanced steatosis. The M2 macrophages were hypothesized to promote apoptosis of M1 polarized hepatic macrophages and protect against disease progression of NAFLD or alcoholic hepatitis (149). Interestingly, soluble CD163 (sCD163), a marker of M1 macrophage polarization, increases systemically with severity of NAFLD in human patients (151), again suggesting that M1 polarization is associated with more advanced stages of the disease.

Traditional treatments for NAFLD are more challenging for elderly populations as they require lifestyle changes in exercise, diet, and medication or liver transplantation (152, 153). Findings from a recent clinical trial using a CCR2/CCR5 antagonist to block inflammation resulted in twice as many subjects achieving improvement in fibrosis and no worsening of steatohepatitis compared with placebo (154, 155). Subgroup analysis showed the therapy to be similarly effective in patients below and above 56 years of age, demonstrating promise in the context of age-associated disease.

## Conclusion

The decline in mitochondrial capacity with aging has emerged as a key mechanism underlying several of the observed changes in the function of both hepatocytes and macrophages, likely contributing to the increased prevalence and severity of chronic liver diseases in elderly populations. In general, the accumulation of infiltrating or M1-polarized monocytes/macrophages tends to exacerbate liver disease and contribute to fibrosis, cirrhosis, and liver failure. However, very few, if any, studies have systematically identified changes in the hepatic macrophage populations of aging livers in either animal models or human patients, representing a hugely understudied area of research.

Liver transplantation is the standard treatment for patients with end stage liver disease. The number of elderly people with liver cirrhosis requiring transplantation has grown over the past 20 years and is expected to increase further (66). Overall survival rates after 1 year are approximately 90% and 10-year survival rates may be more than 70% for recipients (156). However, some studies have found a reduced survival rate in transplant recipients over 60 years of age (from 90 to 64% 1 year survival rate), primarily due to kidney dysfunction and cardiopulmonary disease complications (157). Survival rates improve for elderly patients with few comorbidities (158), thus age alone is not an exclusion criterion from liver transplantation, but all disease risk factors must be considered to devise effective treatment strategies for these age-related chronic liver diseases.

Therapies that suppress M1-polarization or infiltration of macrophages might reduce the progression of liver disease from manageable early stages to chronic end-stages requiring liver transplantation. In the context of aging specifically, promoting M2-gene expression might delay the advancement of liver disease to later stages. However, M2 macrophages have been associated with tumor progression, including clinical hepatocellular carcinoma (HCC) specimens (159). Thus, modulating macrophage polarization has been considered a double-edged sword, and the appearance of liver fibrosis or HCC would need to be closely monitored. Treating the primary drivers of age-associated liver diseases such as alcohol consumption, visceral and ectopic fat accumulation, or deficits in mitochondrial capacity and other age-related mechanisms, will be just as important as targeting the inflammatory symptoms to combat these age-related liver diseases.

Elderly populations continue to increase globally and are particularly at risk for succumbing to liver failure due to the declines in regenerative capacity, reduced survival post-liver transplantation, and tendencies to develop inflammation and fibrosis. Between 1999 and 2016, deaths in the United States from cirrhosis increased by 65% and deaths from HCC doubled (160). A better understanding of the biology of liver aging that influences the onset and progression of chronic liver disease will be crucial in order to develop new interventions and treatments for our aging populations.

## Author contributions

ES, MH, BP, and BB contributed to the writing and editing of the manuscript and figure preparation.

### Conflict of interest statement

The authors declare that the research was conducted in the absence of any commercial or financial relationships that could be construed as a potential conflict of interest.
